# Comparative transcriptomics provides insights into molecular mechanisms of zinc tolerance in the ectomycorrhizal fungus *Suillus luteus*

**DOI:** 10.1093/g3journal/jkae156

**Published:** 2024-07-13

**Authors:** Alexander Smith, Jessica Fletcher, Janne Swinnen, Karl Jonckheere, Anna Bazzicalupo, Hui-Ling Liao, Greg Ragland, Jan Colpaert, Anna Lipzen, Sravanthi Tejomurthula, Kerrie Barry, Igor V. Grigoriev, Joske Ruytinx, Sara Branco

**Affiliations:** Department of Integrative Biology, University of Colorado Denver, Denver, CO 80204, USA; Department of Integrative Biology, University of Colorado Denver, Denver, CO 80204, USA; Research Groups Microbiology and Plant Genetics, Vrije Universiteit Brussel, Ixelles 1050, Belgium; Research Groups Microbiology and Plant Genetics, Vrije Universiteit Brussel, Ixelles 1050, Belgium; Comparative Fungal Biology, Royal Botanic Gardens, Kew, Richmond 11415, UK; Soil, Water and Ecosystem Sciences Department, University of Florida, Gainesville, FL 32351, USA; North Florida Research and Education Center, University of Florida, Quincy, FL 32351, USA; Department of Integrative Biology, University of Colorado Denver, Denver, CO 80204, USA; Centre for Environmental Sciences, Hasselt University, Hasselt 3500, Belgium; DOE Joint Genome Institute, Lawrence Berkeley National Laboratory, Berkeley, CA 94720, USA; DOE Joint Genome Institute, Lawrence Berkeley National Laboratory, Berkeley, CA 94720, USA; DOE Joint Genome Institute, Lawrence Berkeley National Laboratory, Berkeley, CA 94720, USA; DOE Joint Genome Institute, Lawrence Berkeley National Laboratory, Berkeley, CA 94720, USA; Department of Plant and Microbial Biology, University of California Berkeley, Berkeley 94720, CA, USA; Research Groups Microbiology and Plant Genetics, Vrije Universiteit Brussel, Ixelles 1050, Belgium; Department of Integrative Biology, University of Colorado Denver, Denver, CO 80204, USA

**Keywords:** gene expression, metal, zinc, stress, tolerance, fungi

## Abstract

Zinc (Zn) is a major soil contaminant and high Zn levels can disrupt growth, survival, and reproduction of fungi. Some fungal species evolved Zn tolerance through cell processes mitigating Zn toxicity, although the genes and detailed mechanisms underlying mycorrhizal fungal Zn tolerance remain unexplored. To fill this gap in knowledge, we investigated the gene expression of Zn tolerance in the ectomycorrhizal fungus *Suillus luteus*. We found that Zn tolerance in this species is mainly a constitutive trait that can also be environmentally dependent. Zinc tolerance in *S. luteus* is associated with differences in the expression of genes involved in metal exclusion and immobilization, as well as recognition and mitigation of metal-induced oxidative stress. Differentially expressed genes were predicted to be involved in transmembrane transport, metal chelation, oxidoreductase activity, and signal transduction. Some of these genes were previously reported as candidates for *S. luteus* Zn tolerance, while others are reported here for the first time. Our results contribute to understanding the mechanisms of fungal metal tolerance and pave the way for further research on the role of fungal metal tolerance in mycorrhizal associations.

## Introduction

Zinc (Zn) is an essential micronutrient (Robinson *et al.* 2021) critical to biological functions such as enzyme activation and the synthesis of proteins, carbohydrates, and lipids (Sharma *et al.* 2013). However, at high concentrations Zn is toxic and can lead to death (Robinson *et al.* 2021). Alarmingly, increased anthropogenic activity has caused Zn contamination to become one of the most prevalent and detrimental forms of pollution in soil environments (Guo *et al.* 2014, Op de Beeck *et al.* 2015; Smith *et al.* 2016; Ramrakhiani *et al.* 2017). High Zn environments create stressful conditions such as altered soil chemical properties and lower nutrient bioavailability that disrupt soil organism's cellular and organismal processes responsible for metabolism, growth, and survival (Påhlsson 1989; Wang *et al.* 2006; Priyadarshini *et al.* 2021). Despite these harsh conditions, some species tolerate high Zn levels and even thrive in Zn-rich environments (Miransari 2011). The exact mechanisms of Zn tolerance in mycorrhizal fungi are however still unclear.

There has been increasing interest in understanding how fungi interact with Zn (Branco *et al.* 2022). Fungi evolved homeostatic mechanisms to maintain the Zn levels required in cell processes such as transcription, protein folding, and hyphal growth (Feldmann 2012). However, excess Zn negatively impacts fungal cell function by interrupting cell membrane synthesis (Galván Márquez *et al.* 2018), increasing oxidative stress that can trigger programmed cell death (Zadrąg-Tęcza *et al.* 2018), and decreasing growth by disrupting hyphal extension and altering hyphal morphology (Lanfranco *et al.* 2010; Priyadarshini *et al.* 2021). Furthermore, high Zn levels can reduce spore germination (Pawlowska and Charvat 2004), lower fungal survival (Op De Beeck *et al.* 2015; Priyadarshini *et al.* 2021), and decrease fungal community species richness (Faggioli *et al.* 2019).

Even though high Zn concentrations are suboptimal, some fungi evolved high Zn tolerance and can persist in Zn contaminated environments (Ezzouhri *et al.* 2009; Anahid *et al.* 2011; Op De Beeck *et al.* 2015; Robinson *et al.* 2021). Fungal Zn tolerance relies on regulation of Zn transport, sequestration, and immobilization, as well as signaling cascades that trigger stress responses and the production of antioxidants that mitigate toxicity (Bellion *et al.* 2006). Zinc import is controlled by metal transmembrane transporters and the reduction of bioavailable forms of extracellular Zn. For example, in the ericoid mycorrhizal fungus *Oidiodendron maius,* Zn tolerance relies on the plasma membrane Zn transport protein OmFET, and isolates collected from Zn-polluted soils showed a much lower ability to solubilize inorganic Zn (Martino *et al.* 2003; Khouja *et al.* 2013). Additionally, the ectomycorrhizal *Hebeloma cylindrosporum* sequesters cytosolic Zn into intracellular vesicles through a transport protein localized to the endoplasmic reticulum (Blaudez and Chalot 2011). Fungi can also reduce the amount of excess cytosolic Zn through metallothioneins, metal complexing proteins that immobilize micronutrient metals (Howe *et al.* 1997). Finally, antioxidant homeostatic mechanisms produce enzymes that break down reactive oxygen species (ROS), neutralizing oxidative stress toxicity resulting from high intracellular metal content (Bolann and Ulvik 1997; Colpaert 2008; Teng *et al.* 2018).

Studies in *Suillus luteus* have substantially contributed to unveiling the mechanisms of fungal Zn tolerance. This widespread temperate ectomycorrhizal fungus associates with pine trees, maintaining a mutualistic relationship where it provides mineral nutrients and receives carbohydrates in return (Lofgren *et al.* 2024). *Suillus luteus* is an important pioneer species and dominates fungal communities in metal-contaminated sites (Colpaert and van Assche 1987; Ruytinx *et al.* 2011; Op De Beeck *et al.* 2015). This species displays Zn tolerance in areas around decommissioned Zn smelters in Belgium, where some isolates can survive at high Zn soil concentrations (Colpaert and van Assche 1987; Colpaert *et al.* 2004). Notably, Belgian *S. luteus* Zn tolerant isolates accumulate Zn in their tissue at a much slower rate and can withstand much higher concentrations as compared with sensitive isolates (Colpaert *et al.* 2005). Genetic studies on these isolates identified 4 transmembrane transporter genes involved in cellular Zn homeostasis and putatively involved in tolerance. *SlZnT1* and *SlZnT2* are cation diffusion facilitator (CDF) family Zn transporters involved in vacuolar transport, suggesting that intracellular metal sequestration plays an important role in *S. luteus* Zn tolerance (Ruytinx *et al.* 2017). *SlZRT1* and *SlZRT2* are plasma membrane Zrt/IrT-like protein (ZIP) transporters that are downregulated in high Zn environments, indicating they also play a key role in maintaining Zn homeostasis (Coninx *et al.* 2017, 2019). Furthermore, genome scans of the same Belgian *S. luteus* isolates revealed the absence of population structure between isolates from Zn contaminated and noncontaminated soils, and that metal tolerance in this species stems from standing genetic variation and is polygenic (Bazzicalupo *et al.* 2020). Genetically differentiated loci between isolates from contaminated and noncontaminated sites included transmembrane transporters, chelators, and antioxidants, namely, proteins involved in Zn import and vacuolar sequestration, intracellular and extracellular Zn immobilization, and ROS detoxification. Interestingly, the transporters *SlZnT1, SlZnT2*, *SlZRT1*, and *SlZRT2* did not show high genetic divergence (Fst) across isolates from contaminated and noncontaminated sites (Bazzicalupo *et al.* 2020). These results suggested that Zn transporter genes may instead be differentially expressed to achieve Zn tolerance.

Here, we quantify gene expression differences in previously studied Belgian *S. luteus* isolates from contaminated and noncontaminated soils to unveil the mechanisms of fungal Zn tolerance. We hypothesized that isolates originating from contaminated soils would be Zn tolerant, and isolates from noncontaminated soils would be sensitive to high Zn concentrations. Because these isolates belong to the same population and are genetically very similar (Bazzicalupo *et al.* 2020), we expected to find significantly different transcriptomic profiles only across high- and low-Zn treatments, including in the candidate genes highlighted in previous experiments. We also predicted that tolerant and sensitive isolates would show distinct responses to the Zn treatment, with differentially expressed genes contributing to Zn tolerance pathways and mechanisms in *S. luteus*. We found that the level of soil contamination was positively associated with isolate Zn tolerance and that transcriptomic differences between tolerant and sensitive isolates were mainly constitutive. We also document *S. luteus* putative Zn tolerance mechanisms, including metal exclusion and immobilization, as well as recognition and mitigation of metal-induced oxidative stress. Our analyses provide a transcriptome-wide exploration of potential ectomycorrhizal fungal Zn tolerance mechanisms and highlight many genes likely important to Zn tolerance in *S. luteus.*

## Materials and methods

### Sampling sites and fungal culture information

To examine the effect of Zn on *S. luteus* gene expression, we investigated 10 previously studied isolates from Belgium, 5 collected from 1 metal-contaminated site (C1-C5) and 5 collected from 1 noncontaminated site (NC1-NC5, Colpaert *et al.* 2004, Supplementary Table 1). All 10 isolates show little overall genetic differentiation and belong to a single population (Bazzicalupo et al 2020). Both contaminated and noncontaminated sites are dominated by Scots pine (*Pinus sylvestris*), and the contaminated site displays high levels of heavy metals resulting from now decommissioned zinc smelters (Sonke *et al.* 2002; Op De Beeck *et al.* 2015). The soil Zn content was previously determined at a maximum of 1,750μg/g dry weight for the contaminated site and 21 μg/g dry weight for the noncontaminated site (Colpaert *et al.* 2000). We obtained *S. luteus* pure cultures from fruit bodies and maintained them in solid modified Fries medium (Colpaert *et al.* 2000; Lofgren *et al.* 2024). For more information on collection methods and site descriptions, consult Bazzicalupo *et al.* (2020) and Colpaert *et al.* (2000).

### Testing *S. luteus* Zn tolerance

To quantify *S. luteus* Zn tolerance and determine whether it is associated with soil contamination, we calculated EC_50_ values (the amount of Zn that inhibits isolate growth by 50%) for each of the 10 isolates. We grew the isolates at 23°C in darkness on cellophane-covered solid modified Fries medium supplemented with different concentrations of ZnSO_4_·7H_2_O (0, 100, 200, 400, 800, and 1,000 ppm). We performed 4 replicates per isolate for each condition. After 14 days of growth, we collected the mycelium from the plates and stored it at −80°C. To determine EC_50_ values per isolate, we followed the protocol in Ritz et al. (2015). Briefly, we obtained isolate dry weight after lyophilization and used it to construct dose–response curves through nonlinear regression with a 4-parameter log-logistic model in R version 3.5.1 (R Core Team 2018; RStudio Team 2019). We used EC_50_ values to place isolates into tolerance categories (tolerant, sensitive) irrespective of collection site. We considered isolates Zn tolerant if EC_50_ > 130 ppm and sensitive if EC_50_ < 130 ppm as this was the amount of Zn added in our transcriptomic experiment (see details in the following).

### RNA extraction and sequencing

To measure Zn-induced differential gene expression across the Zn-tolerant and Zn-sensitive *S. luteus* isolates, we grew the isolates on both control and zinc-supplemented (130 ppm Zn) modified solid Fries medium covered with cellophane, in triplicate, at 23°C, and in darkness (*n* = 10 isolates × 2 treatments × 3 replicates = 60, Supplementary Table 2). We selected 130 ppm Zn because this concentration stresses but does not kill sensitive isolates and allows for comparing gene expression across Zn-tolerant and -sensitive *S. luteus*. Once the isolate on the control plate reached a diameter of 3 cm (10–14 days, depending on the isolate), the mycelium for both control and corresponding Zn supplemented plates was collected and stored at −80°C awaiting RNA extraction.

We extracted total RNA using the RNeasy Plant Mini Kit (Qiagen, France) on mycelia ground in liquid nitrogen using mortar and pestle. We measured the RNA concentration and determined the A260/230 and A260/280 ratios using a Nanodrop One (Thermo Fisher). Samples that did not meet purity criteria (A260/230 and A260/280 > 1.8) were discarded. RNA integrity was confirmed using a Bioanalyzer with the RNA 6000 Nano Kit (Agilent). Two sample replicates (one NC1 and one NC3, both grown in 130 ppm Zn) did not meet our quality criteria and were not incorporated into subsequent analyses. RNA was sequenced by the Joint Genome Institute (JGI) on an Illumina NovaSeq 6000 S4 (2 × 151 bp) and run through JGI's quality and quantity quality control tests (https://jgi.doe.gov/user-programs/pmo-overview/project-materials-submission-overview/). Three sample replicates did not meet sequence quality standards (2 from isolate NC2 and 1 from isolate NC5 all in 130 ppm Zn) and were discarded, so that our final number of samples for gene expression analysis was reduced to 55. Supplementary Table 2 lists the Short Read Archive codes for all samples.

### Bioinformatics pipeline

The RNA sequence analyses involved 5 steps: read quality control, alignment to reference genome and raw gene counts, differential gene expression analyses, gene ontology (GO) analyses, and co-expression network construction:

#### Read quality control and preprocessing

We used BBDuk (Bushnell 2014) to filter and trim raw reads. We used kmer matching (kmer = 25) to remove Illumina adapters, sequencing artifacts, RNA spike-in reads, PhiX reads, and reads containing any ‘N's. We used the phred trimming method to trim the read ends, set to Q6. Finally, we removed reads shorter than 50 bases (1/3 the length of the original read). Supplementary Table 3 lists all sample raw and filtered read counts.

#### Alignment to reference genome and raw gene counts

We used HISAT2 (Kim *et al.* 2015) to align reads to the *S. luteus* reference genome, calculate gene counts, and extract gene annotations. The reference genome (*S. luteus* isolate UH-Slu-Lm8-n1, https://mycocosm.jgi.doe.gov/Suilu4/Suilu4.info.html) originated from the same Belgian population as the isolates used in our study (JGI Project ID: 1006871; Kohler *et al.* 2015). To calculate raw gene counts, we used the program featureCounts (Liao *et al.* 2014) and the gff3 annotation file from the reference genome. Specifically, we only used primary hits on the reverse strand for the final gene counts (-s 2 -*P* –primary options).

#### Differential gene expression analysis

To characterize the differences in transcriptomic response to Zn between tolerant and sensitive isolates, we fit a generalized (negative binomial) linear model using the R package DESeq2 (Love *et al.* 2014) with the 55 retained RNAseq sample counts per gene as input and including parameters for Zn treatment (discrete factor, 2 levels, high, or control), isolate (discrete factor, 10 levels, NC1-5, and C1-5), and the interaction between these 2 effects. We then built 5 contrasts, (1) Zn treatment contrast: high Zn—control Zn, (2) tolerance group contrast: tolerant isolates—sensitive isolates, (3) tolerant Zn contrast: tolerant high Zn—tolerant control Zn, (4) sensitive Zn contrast: sensitive high Zn—sensitive control Zn, and (5) interaction contrast: tolerant Zn contrast—sensitive Zn contrast, outputting DESeq results tables for each comparison. To account for errors in modeling, we calculated a Benjamini–Hochberg (false discovery rate, FDR) adjusted *P*-value for each transcript using Wald tests and used a threshold of FDR-adjusted *P*-value < 0.05 as adequate evidence that a transcript was differentially expressed. We then used the results tables and associated transcript FDRs to identify 3 groups of interest: (1) transcripts that were differentially expressed in response to the Zn treatment irrespective of the sensitivity of the isolate (Zn treatment FDR < 0.05; interaction FDR > 0.05), (2) transcripts that were differentially expressed between tolerant and sensitive isolates irrespective of Zn treatment (tolerance group FDR < 0.05, interaction FDR > 0.05), and (3) transcripts whose differential expression in response to Zn treatment differed between tolerant and sensitive isolates (interaction FDR < 0.05). Importantly, these categories allow us to interpret the direction and magnitude of differential expression in a biologically relevant manner. Our approach to DESeq analysis, first retaining isolate distinction in our model as a parameter, and then incorporating tolerance groups into the contrasts when outputting the results tables, is an attempt to account for isolate-to-isolate variation, since DESeq2 has no framework to model the random effect of isolates within tolerance groups. We have also applied additional analyses in the following that allow visualization and inference about variation among isolates.

To visualize clustering of samples based on overall transcript expression in the model, we used the *plotPCA* function of DESeq2 to run a principal component analysis (PCA). To test for differences between treatment groups in the model, we ran a PERMANOVA with a pairwise distance matrix, calculated using the Jaccard index, as input and including parameters for Zn treatment (discrete factor, 2 levels, high or control), tolerance group (discrete factor, 2 levels, tolerant and sensitive), and the interaction between these 2 effects (permutations = 9,999, vegan package: Oksanen *et al.* 2024). To account for the large number of permutations in the PERMANOVA, we then used the Benjamini–Hochberg method to adjust all *P*-values. To visualize differential expression of Zn response for each individual isolate, we made volcano plots by first classifying all DESeq input data into subsets by isolate and then conducting DESeq analyses separately for each isolate. We then used the R package EnhancedVolcano (Blighe *et al.* 2024) to create volcano plots for all 10 isolates (Supplementary Fig. 4 in Supplementary File 1). To visualize overall differential expression across all replicates, we made correlation matrices of the normalized counts for all RNAseq samples in the control (*n* = 30) and high Zn conditions (*n* = 25) using the base R function *cor* with the Pearson method. We then used the *heatmap.2* function in the gplots package (Warnes *et al.* 2024) to plot the correlation matrices (Supplementary Fig. 7 in Supplementary File 1). To obtain predicted functions of differentially expressed genes, we used the search function of JGI MycoCosm genome portal (Ashburner *et al*. 2000; Nordberg *et al*. 2014; The Gene Ontology Consortium *et al*. 2023) on the genome Suillus luteus UH-Slu-Lm8-n1 v3.0 (Kohler *et al*. 2015).

#### Gene ontology enrichment analysis

To identify enriched (overrepresented) gene annotation categories in the 3 groups of transcripts, we implemented an enrichment analysis using ClueGO (v2.5.9) (Bindea *et al.* 2009), a Cytoscape (Shannon *et al.* 2003) application. This analysis used the GO annotation database to extract gene annotation categories that were enriched in each of the transcript groups as compared to the whole genome. We investigated GO annotation terms from all 3 overarching categories (biological process, molecular function, and cellular component) and used modified settings for both the GO Tree Interval (min = 3; max = 15) and GO term/pathway selection (min # genes = 1; % genes = 4) to include the most number of GO terms as possible. We used 2-sided hypergeometric tests with a Benjamini–Hochberg (FDR) correction to weight evidence for enrichment, reporting only GO terms with an FDR-adjusted *P*-value ≤ 0.05. Additionally, to identify highly differentially expressed genes, we restricted our analysis to transcripts only with an absolute log2-fold change value >1.

#### Co-expression network construction

To determine modules of genes that were highly correlated with either Zn treatment or with EC_50_ value, we conducted weighted gene co-expression network analysis using the WGCNA R package v 1.72-5 (Langfelder and Horvath 2008). Prior to performing this analysis, we excluded 2 samples (NC3 with Zn and without Zn) as they were significant outliers in clustering analysis (Supplementary Fig. 1 in Supplementary File 1). We performed signed network construction and module detection using the *blockwiseModules* function, a module cut height of 0.25, and a soft thresholding power of 16, selected after examining a range of powers for *R*^2^ > 0.8 and minimal mean connectivity (Supplementary Fig. 2 in Supplementary File 1). This generated a list of module eigengenes, and we used the *plotEigengeneNetworks* function to generate a heatmap of adjacencies among eigengenes and a dendrogram describing their relationships (Supplementary Fig. 3 in Supplementary File 1). Module eigengenes are the first principal components of each module and are representative of the gene expression profile of the module.

We used the *labeledHeatmap* function to investigate the correlation between modules and samples traits (Zn treatment or EC_50_ value). For each trait, we selected modules that were significantly correlated with the trait and plotted the module membership statistic against the gene significance statistic to determine which modules displayed a strong positive correlation (i.e. genes with importance within the module that was correlated with significance to the trait). Modules with a significant positive correlation were selected for GO analysis. Genes within the modules, along with their trait significance *P*-value were subjected to GO analysis using the topGO R package v 3.18 (Alexa and Rahnenfuhrer 2024 Alexa & Rahnenfuhrer 2023). We examined GO terms and visualized results using ggplot2 (Wickham *et al.* 2024).

We also performed intermodular analysis to examine gene significance to either trait (Zn treatment or EC_50_ value) across all genes in the dataset. Each list of genes was ranked based on the –log10 of the gene trait significance *P*-value, and was subjected to preranked gene set enrichment analyses (Mootha, *et al.* 2003; Subramanian *et al.* 2005) using a gene matrix file consisting of GO terms (biological process, molecular function, and cellular component) and their corresponding genes.

## Results

### *Suillus luteus* Zn tolerance is associated with soil contamination

**Fig. 1.**
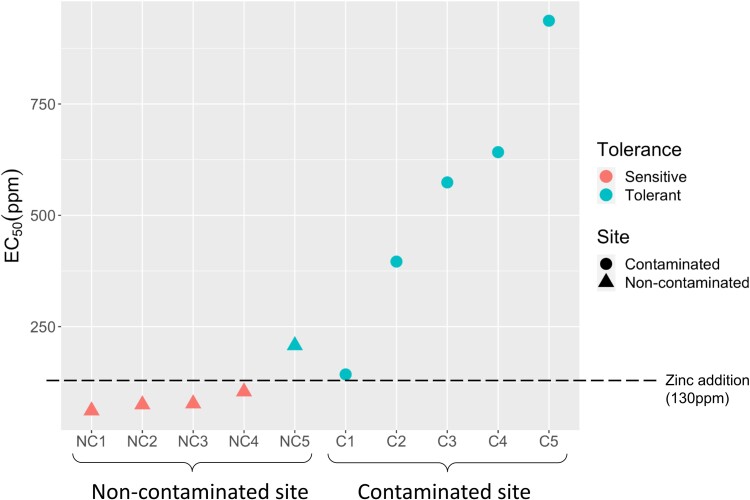
*Suillus luteus* Zn tolerance and soil contamination. EC_50_ values for the studied *S. luteus* isolates, grouped by collection site (NC, noncontaminated site; C, contaminated site). The shape of the symbols denotes collection site (triangles, noncontaminated site, circles, contaminated site), and the color of the symbols denotes tolerance group. Dashed line indicates 130 ppm Zn, the amount of Zn added to the Zn-treated plates.

We found *S. luteus* Zn tolerance is strongly associated with soil contamination (ANOVA: *F*_1,8_ = 10.38, *p* = 1.22e^−02^). Isolates collected from the contaminated site were more Zn tolerant than isolates collected from the noncontaminated site, with the most tolerant isolate (C5) having an EC_50_ of 937 ppm and the most sensitive isolate (NC1), 61 ppm (Fig. 1; Supplementary Table 1). Interestingly, one isolate from the noncontaminated site was more Zn tolerant than one of the isolates from the contaminated site (Fig. 1; Supplementary Table 1). The 4 sensitive isolates had EC_50_ values lower than the Zn concentration used in the experiment (130 ppm), but were able to survive the treatment allowing us to measure gene expression. Conversely, for most Zn tolerant isolates, EC_50_ concentration was considerably greater than the amount of added Zn and may not have been as affected by the Zn addition. Based on these EC_50_ values, we divided the 10 isolates into Zn-tolerant and -sensitive groups (Fig. 1) as detailed in the Methods.

### Zn-tolerant *S. luteus* isolates show higher overall transcriptomic variation compared with sensitive isolates

**Fig. 2.**
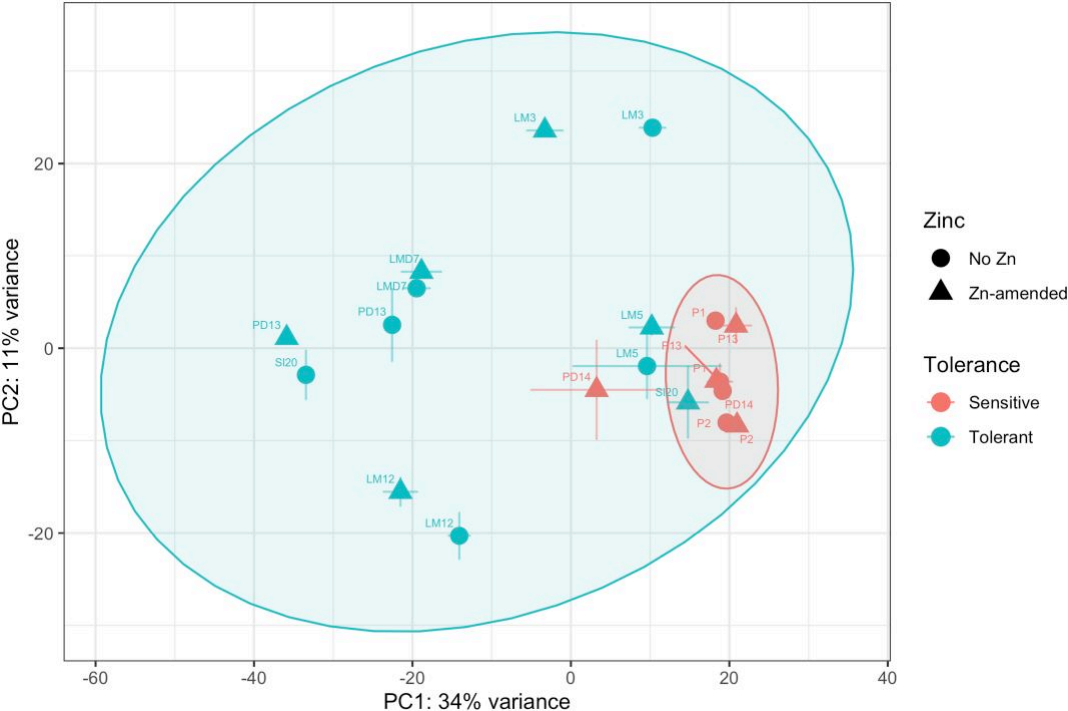
Tolerant isolates show higher transcriptomic variation compared to sensitive isolates. Principal component analysis plot of complete transcriptomes of Zn-sensitive and -tolerant *S. luteus* isolates. Symbols represent the average across isolate replicates, and error bars represent the standard error between replicates. Colors denote tolerance grouping and the symbol shape denotes Zn treatment (circle, no zinc, triangle, zinc amended). We include isolate identifiers next to each symbol. Ellipses denote 95% confidence intervals for each of the tolerance groups (red, sensitive, blue, tolerant).

The PCA showed that *S. luteus* isolates displayed significant transcriptomic variation across Zn-tolerance groups (PERMANOVA: *F*_1,51_ = 4.98, *P*-adj = 3.00*e*^−04^) and Zn treatment (PERMANOVA: *F*_1,51_ = 2.71, *P*-adj = 3.45*e*^−03^) (Fig. 2). Notably, the different Zn-tolerant isolates showed higher variation in gene expression profiles compared to sensitive isolates (Fig. 2). We did not find a significant tolerance group-dependent Zn effect (interaction effect) (PERMANOVA: *F*_1,51_ = 1.06, *P*-adj = 0.366), indicating that tolerant and sensitive isolates do not show significantly different overall transcriptomic variation under Zn exposure. There were 2 exceptions to this pattern, with isolate C3 showing large differences in differential gene expression across Zn treatments (Fig. 2; Supplementary Fig. 4 in Supplementary File 1) and C4 showing transcriptomes more similar to sensitive isolates, independently of Zn treatment (Fig. 2).

### Isolate Zn tolerance level, not Zn treatment, drives the number of differentially expressed genes

**Fig. 3.**
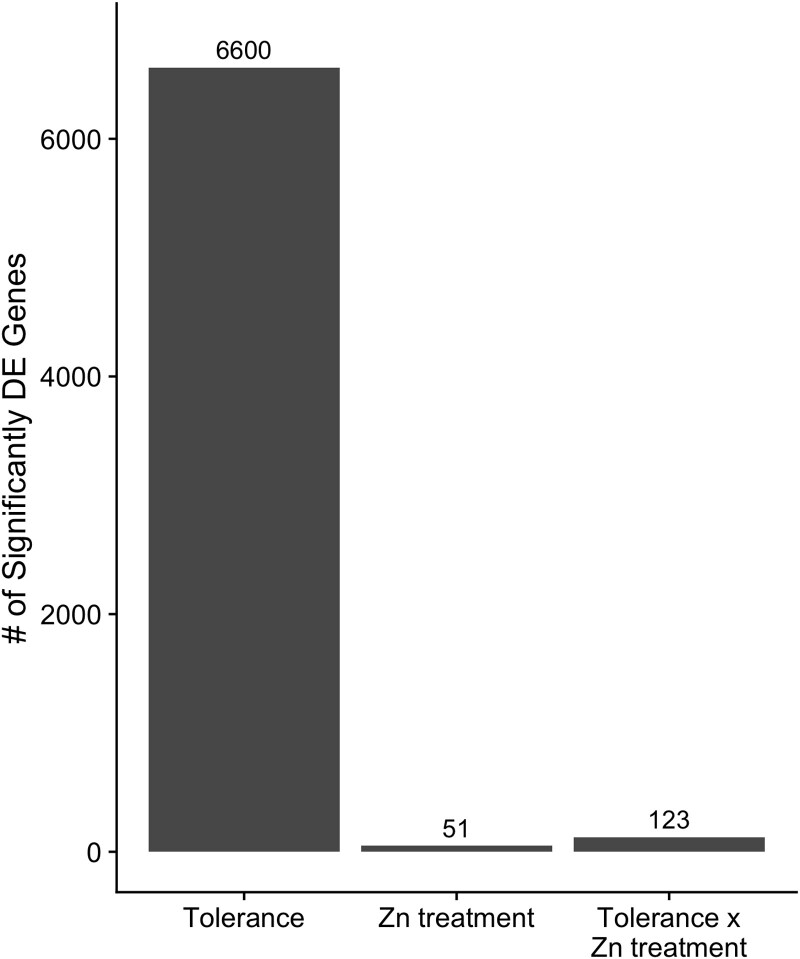
Zn tolerance affects gene expression more than Zn treatment. Barplot displaying the number of significantly differentially expressed (DE) genes in each of the three model comparisons. Tolerance = tolerance groups (tolerant vs sensitive), Zn treatment = control vs Zn-amended, Tolerance × Zn treatment = interaction effect. The numbers on top of bars represent the number of significantly DE genes. DE genes that had a significant interaction effect were removed from the DE gene counts for the main effects (Tolerance and Zn treatment).

The differential gene expression analysis showed that the vast majority of genes (6,600) were differentially expressed between isolate Zn tolerance groups and that Zn treatment induced a very small number of differentially expressed genes (51) (Fig. 3). Specifically, we found ∼130 times as many genes were significantly differentially expressed between tolerant and sensitive isolates as compared to Zn treatment. In addition, we only detected 123 genes for which tolerant and sensitive isolates responded to Zn differently (interaction effect; Fig. 3).

### Enrichment of transmembrane transport, oxidoreductase, protein kinase, and fungal hydrophobin activities

**Fig. 4.**
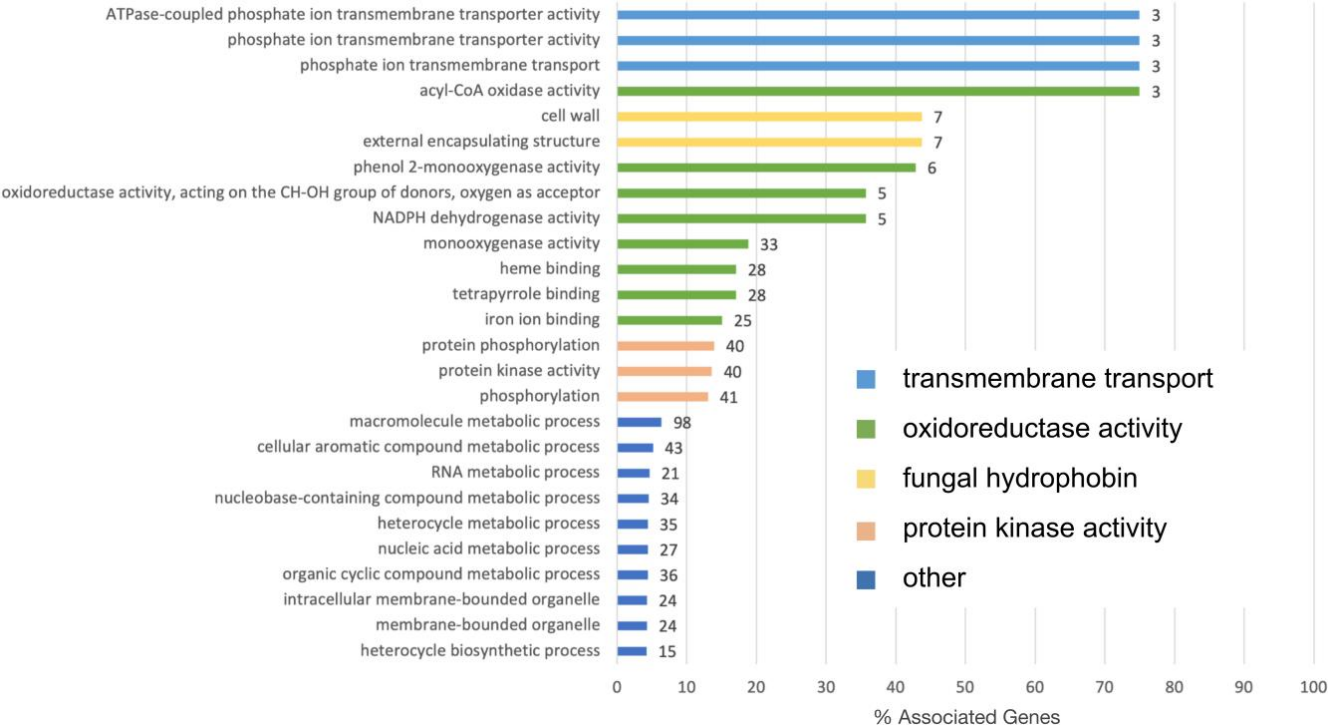
Significantly differentially expressed genes between tolerant and sensitive isolates are enriched in transmembrane transport, oxidoreductase, fungal hydrophobin, and protein kinase activity. Barplot showing enriched GO terms in significantly differentially expressed genes between tolerant and sensitive isolates. Bar length represents the percentage of all genes with the GO term annotation that appear in this gene group. The numbers at the end of the bars represent the number of unique genes with that GO term annotation. Colors represent general functional categories (light blue, transmembrane transport; green, oxidoreductase activity; yellow, fungal hydrophobin; orange, protein kinase activity; and dark blue, other).

We found 26 enriched GO terms in the response to tolerance group and no enriched GO terms in the response to Zn treatment or interaction effect. There was a large overlap in the functions of enriched GO terms in genes differentially expressed between tolerant and sensitive isolates, so we merged them into 4 categories: transmembrane transport, oxidoreductase activity, fungal hydrophobins, and protein kinase activity. Transmembrane transport was the most enriched annotation category (highest % associated genes) and corresponded to three pleiotropic drug resistance (PDR) transporters (Fig. 4).

### Significant differential expression of candidate genes between Zn-tolerant and -sensitive isolates irrespective of Zn treatment

Most previously identified *S. luteus* Zn tolerance candidate genes, namely, Zn transporters and genes genetically diverged between isolates from contaminated and noncontaminated sites (Coninx *et al.* 2017, 2019; Ruytinx *et al.* 2017; Bazzicalupo *et al.* 2020), showed significant differential expression only across tolerance groups (Table 1). Specifically, 7 Zn transport genes, 3 metal transport genes, 3 chelators, and 4 oxidative stress relief genes were differentially expressed between tolerant and sensitive isolates irrespective of Zn treatment (tolerance group FDR < 0.05, interaction FDR > 0.05; Table 1). None of the candidate genes showed significantly different gene expression across Zn treatment, irrespective of tolerance group (Zn treatment FDR < 0.05, interaction FDR > 0.05; Table 1), or interaction between tolerant isolates and Zn treatment (Table 1), and sensitive isolates and Zn treatment (Table 1) (interaction FDR > 0.05). Of the 17 differentially expressed genes across tolerance group, 12 were upregulated in tolerant isolates as compared with sensitive isolates and 5 genes were upregulated in sensitive isolates as compared with tolerant isolates. The most upregulated candidate gene in the tolerant isolates was a cytochrome P450, a detoxification agent, while in the sensitive isolates, it was a fungal hydrophobin, a metal chelator.

**Table 1.** Differential expression of previously identified S. luteus Zn transporters and genetically diverged genes only across tolerant and sensitive isolates.

	Gene category	Predicted/potential function (citation)	Annotation	Protein ID	(A) Tolerance groups Log2-fold change	(B) Zn treatment Log2-fold change	(C) Tolerant Zn treat Log2-fold change	(D) Sensitive Zn treat Log2-fold change
Zn transporters	Cation diffusion facilitator (CDF) transporter	Zinc transporter from cytosol into vacuole (Ruytinx *et al.* 2017)	Cation Diffusion facilitator (CDF) transporter (SlZnT1)	2846331	0.3216**	−0.0025	−0.0514	0.0710
		Zinc transporter from cytosol into cellular compartment or extracellular space (Ruytinx *et al.* 2017)	Cation diffusion facilitator (CDF) transporter (SlZnT2)	2854961	−2.8822*	−0.0965	−0.0315	−0.1940
		Zinc transporter from cytosol into vesicle/vacuole (Eide 2006)	ZRG17-like CDF transporter (SlCDF-B)	2859797	0.2189**	−0.0599	−0.0920	−0.0118
			MMT1-like CDF transporter (SlCDF-C)	810602	0.2526**	−0.0252	0.0860	−0.1920
			Cation diffusion facilitator (CDF) transporter (SlCDF-D)	72605	−8919*	−0.0386	0.1204	−0.2769
	ZRT–IRT-like protein (ZIP) transporter	Zinc importer from extracellular space into cytosol (Coninx *et al.* 2017)	ZRT–IRT-like protein (ZIP) transporter (SlZRT1)	2764984	−0.0488	−0.2248	−0.8986	0.7859
		Zinc importer from extracellular space or cellular compartment into cytosol (Coninx *et al.* 2019)	ZRT–IRT-like protein (ZIP) transporter (SlZRT2)	2893674	0.0365	−0.0535	−0.2365	0.2210
		Zinc transporter from extracellular space into cytosol (Eide 2006)	ZRT–IRT-like protein (ZIP) transporter (SlZIP-C)	2856429	0.2834**	−0.1318	−0.1678	−0.0779
			ZRT–IRT-like protein (ZIP) transporter (SlZIP-D)	1739397	0.2599**	−0.0337	−0.0606	0.0066
Genetically diverged genes	Metal ion transporters	Zinc binding site (Zhang *et al*. 2000)	Rab geranylgeranyltransferase	2921934	0.6166**	0.0528	0.0254	0.0940
		Zinc transporter from cytosol into vesicle/vacuole (Eide 2006)	Cation diffusion facilitator (CDF) transporter	2898571	0.1052**	−0.1081	−0.1569	−0.0350
		Iron transporter from cytosol into vesicle/vacuole (Ferrol *et al.* 2016)	Iron permease (FTR1)	2861746	1.3131**	−0.2802	−0.1201	−0.5202
	Chelating agents	Synthesis of nicotianamine, a cadmium chelating agent in cytosol (Aloui *et al*. 2011)	S-adenosyl-L-methionine-dependent methyltransferase	2848300	0.0162	0.1133	0.2791	−0.1353
				2854576	0.1806**	0.0056	0.0565	−0.0707
				2861857	0.0812	0.0114	0.0501	−0.0466
		Response to stress and chelating secreted protein (Ferrol *et al.* 2016)	Heat shock protein 70 family	2852980	−0.2071*	−0.0615	−0.0220	−0.1208
		Metal ion chelating secreted protein (Ferrol *et al.* 2016)	Fungal hydrophobin-domain-containing protein	2921647	−4.1776*	−0.2140	−0.1163	−0.3606
	Oxidative stress relief	Antioxidant (Schroder *et al*. 2003)	Flavin Adenin dinucleotide (FAD)-binding	2849043	0.4427**	0.0045	0.0243	−0.0253
			Ferric reductase NAD binding	2623337	0.7628**	0.0564	−0.0373	0.1969
		Pollution detoxification (Van Den Brink *et al*. 1998)	Cytochrome P450	2722447	3.6920**	1.7388	1.3288	2.3538
			Cytochrome P450	83946	−2.6008*	0.0753	0.1232	0.0034

Differential expressions across model comparisons of previously identified genes putatively involved in *S. luteus* metal tolerance. Log2-fold change (lfc) values display differential gene expression in response to tolerance groups (A), Zn treatment (B), Zn treatment in tolerant isolates only (C), and Zn treatment in sensitive isolates only (D). Colored log2-fold change values have an adjusted *P*-value of < 0.05 and denote the direction of differential expression (*light gray = lfc < 0, **dark gray = lfc > 0).

### Many other genes were differentially expressed across comparisons

We found many other differentially expressed genes across each of the three model comparisons that have not been highlighted in previous studies. Of the genes with the most positive and negative log2-fold change values in the three model comparisons (*n* = 58), close to 70% had no annotation (*n* = 39; Supplementary Table 4). The remaining annotated genes had predicted functions relating to processes such as nucleic acid metabolism (9 genes), signal transduction (2 genes), and enzyme activity (4 genes). Genes that were differentially expressed in response to Zn treatment had predicted functions related to signal transduction, enzyme activity, and the regulation of nucleic acids (Supplementary Table 4).

### Co-expression network analyses show cellular signaling, oxidoreductase, metal ion binding, and transporter activity are important in *S. luteus* Zn response and tolerance

**Fig. 5.**
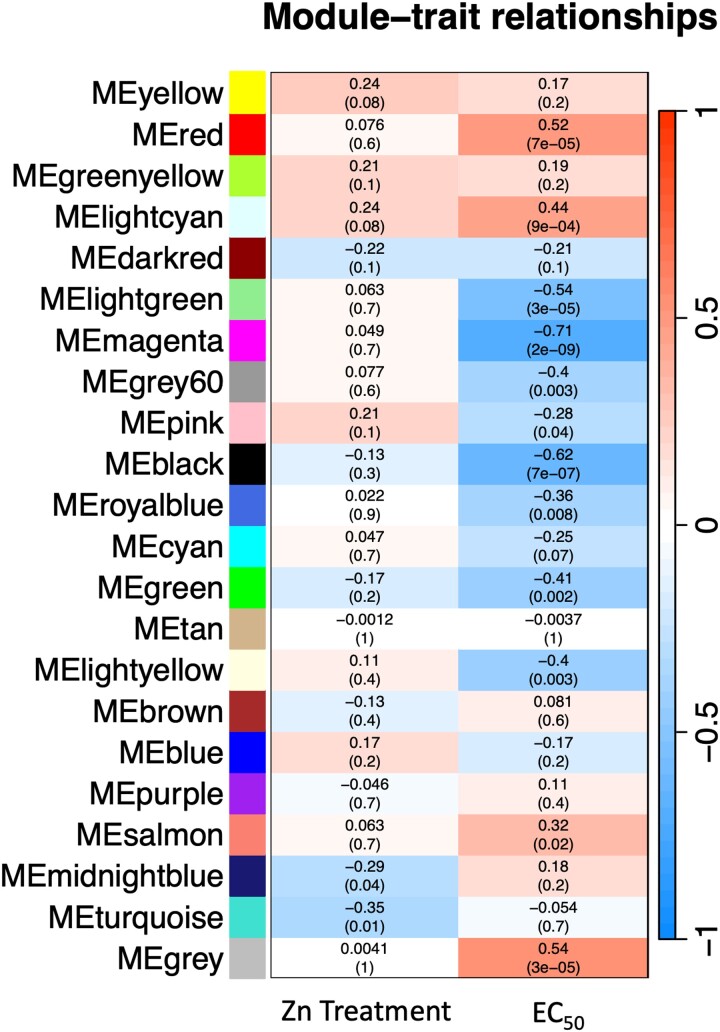
Module–trait associations determined by Pearson correlation. The numbers in each cell indicate the correlation between the trait (columns) and the modules (rows), with the color scale representing positive (red) to negative (blue) correlation. Correlation *P*-values are in parenthesis. The Zn treatment trait is a binary representation of whether Zn treatment was applied (1) or not (0), while the EC50 trait is the numerical value for tolerance (higher value = more tolerant).

The WGCNA detected modules of genes significantly associated with either Zn treatment or EC50 (Supplementary Fig. 5 in Supplementary File 1). Two gene modules (midnightblue and turquoise) were significantly negatively correlated with Zn treatment (Fig. 5), indicating that Zn addition decreases expression of the module eigengenes. The turquoise module was significantly enriched for molecular function, cellular compartment, and biological process GO terms and had 18 genes predicted to be transcription factors (Supplementary Tables 5 and 6). The midnightblue module was not significantly enriched for any GO terms and had one gene predicted to be a transcription factor (Supplementary Table 6).

We found three modules (red, lightcyan, and salmon) significantly positively correlated with EC_50_ value (with expression of genes within the modules increasing with EC_50_) and eight modules (lightgreen, magenta, grey60, pink, black, royalblue, green, and lightyellow) significantly negatively correlated with this trait (with expression of genes within the modules decreasing with EC_50_) (Fig. 5). Out of these modules, only the lightgreen, magenta, black, and green showed significant positive correlation between module membership (importance of the gene within the module) and gene significance for EC_50_ value (importance of gene to the trait). The black module was significantly enriched for GO:0016614 oxidoreductase activity that acts on CH–OH group of donors. The green module was only significantly enriched for GO:0016740 transferase activity and GO:0003824 catalytic activity. The magenta and lightgreen modules were not significantly enriched for any GO terms. We found genes predicted as transcription factors within each module, specifically 3 in the black module, 7 in the green module, 3 in the magenta module, and 1 in the lightgreen module (Supplementary Table 6).

Intermodular analysis (investigating genes significantly associated with traits irrespective of modules) identified genes correlated to either Zn treatment or to EC_50_ (Supplementary Fig. 6). For Zn treatment, gene set enrichment analyses highlighted gene sets involved in intracellular signaling (GO:0007242), metal ion binding (GO:0046872), and metal ion transport (GO:0030001) (Supplementary Table 7). The last included two previously identified *S. luteus* Zn transporters, *SlZIP-B* (2893674) and *SlZRT1* (2764984). For EC_50_, we found enrichment for gene sets involved in oxidoreductase activity (GO:0016614), metal ion binding (GO:0046872), and cation transport (GO:0006812) (Supplementary Table 7). The last included previously described *Suillus* Zn binding genes *SlZnT2* (2854961), *SlZnT1* (2846331), and *SlCDF-D* (72605).

## Discussion

**Fig. 6.**
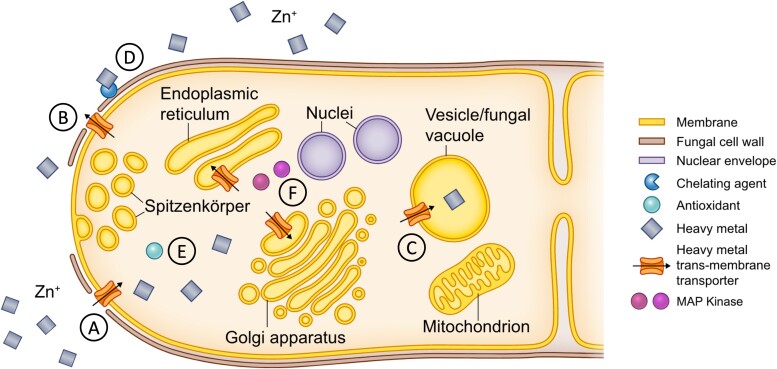
Potential mechanisms of Zn tolerance in *S. luteus.* Diagram of putative mechanisms of Zn tolerance in *S. luteus*. (A–C) Zn transmembrane transport including Zn importers (such as ZIP transporters), exporters (such as CDF transporters), and sequestration transporters (such as CDF and PDR genes), which maintain cytosolic Zn homeostasis. (D) Zinc chelation (for example, through fungal hydrophobins), which immobilizes excess Zn. (E) Antioxidants that mitigate metal-induced oxidative stress. (F) MAP kinases that transmit stress signals to the nucleus. (Adapted from Branco *et al.* 2022).

We investigated Zn tolerance and differential gene expression in *S. luteus* isolates collected from metal-contaminated and noncontaminated sites. We found that in this species Zn tolerance is associated with soil contamination, is both a constitutive and environmentally dependent trait, and results from a combination of responses involving metal exclusion and immobilization, as well as recognition and mitigation of metal-induced oxidative stress (summarized in Fig. 6).

The *S. luteus* Zn-tolerant isolates were mainly associated with high soil Zn content, corroborating previous research (Colpaert et al 2004). However, one isolate from the contaminated site showed Zn tolerance more similar to isolates from the noncontaminated site. This discrepancy likely results from soil heterogeneity and the existence of low-Zn pockets within the contaminated site, allowing the persistence of Zn-sensitive isolates. In fact, the soil Zn dry weight concentrations at the contaminated site can be as low as 1 ppm (Op De Beeck *et al.* 2015), making it likely for Zn-sensitive fungi to be able to survive in localized areas of low-Zn concentration. Another possibility is that soil Zn bioavailability varies across the contaminated site and that Zn can locally occur in a form that is not toxic to fungi, allowing the sensitive isolate to persist.

The *S. luteus* Zn tolerant and sensitive isolates had markedly different overall transcriptomic profiles, with tolerant isolates displaying much higher overall transcriptomic variation and few individual genes being differentially expressed in response to Zn treatment. This means that Zn tolerant isolates consistently maintain a Zn-tolerant gene expression profile. Constitutive expression independent of external Zn concentration was previously documented for two *S. luteus* Zn transmembrane transporters (Ruytinx *et al.* 2017). Here we show that it applies to a much larger number of genes, including functions such as cation transport and oxidoreductase activity. The large number of observed gene expression differences across tolerant isolates also suggests the existence of distinct paths to achieve Zn tolerance in the species, through the expression of different sets of metal tolerance-related genes.

While constitutive tolerance mainly explains differences between tolerant and sensitive isolates, tolerant isolates also display Zn-induced gene expression, although on a much smaller scale. For example, WGCNA showed Zn transporters were involved in Zn response regardless of tolerance level and that the previously identified transporters *SlZIP-B* (2893674) and *SlZRT1* (2764984) were significantly differentially regulated when isolates were exposed to Zn. Interestingly, Zn transporters implicated in the environmental response were different than those involved in constitutive tolerance.

Zn tolerance in *S. luteus* is associated with a clear enrichment of metal homeostasis-related genes, including transmembrane transport, oxidoreductase, fungal hydrophobin, and protein kinase activity. Genes involved in signaling and metal transport, immobilization, and detoxification have been previously implicated in metal response (Wolfger *et al.* 2001; Lin *et al.* 2005; Bazzicalupo *et al.* 2020). In *S. luteus*, enriched transmembrane transport activity corresponded to PDR transporters, ATP-binding cassette transporters that have been linked to metal stress in both plants and fungi (Wolfger *et al.* 2001; Nuruzzaman *et al.* 2014). In yeast, PDR transporters are involved in vacuolar sequestration of chelated metal conjugates (Buechel and Pinkett 2020) and could have a similar function in *S. luteus* (Fig. 6). Oxidoreductase activity corresponded to genes in a variety of pathways that catalyze electron transfer between molecules (Bolann and Ulvik 1997). Oxidative stress is a known byproduct of metal exposure and is characterized by the accumulation of ROS (Azevedo *et al.* 2007; Liang and Zhou. 2007), which can impair cellular functioning and result in cell death. The reduction of ROS by oxidoreductase activity is an important homeostatic response (Zadrąg-Tęcza *et al.* 2018). Fungal hydrophobin activity corresponded to cell wall fungal hydrophobin genes that function by binding excess metals to render them inert (Ferrol *et al.* 2016; Fig. 6) and have been implicated in the metal response of mycorrhizal fungi (Sammer *et al.* 2016). Several genes involved in protein kinase activity were predicted mitogen-activated protein kinases, evolutionary conserved signal transduction modules that convert environmental cues into cell responses (Mishra *et al.* 2006; Cristina *et al.* 2010). These proteins have been shown to be activated in response to metal stress (Lin *et al.* 2005; Mondal 2022), to function in the signal cascade mechanism of metal homeostasis (Mondal 2022), and likely play an important role in *S. luteus* Zn tolerance (Fig. 6).

Differential expression of candidate genes putatively involved in Zn tolerance across *S. luteus* further confirms marked differences across Zn-sensitive and -tolerant isolates, corroborates constitutive Zn tolerance in this species, and hints at potential *S. luteus* tolerance mechanisms. Zn tolerance in this species is mainly achieved through constitutive differential expression of candidate metal ion transporters, metal chelating agents, and antioxidants (Bazzicalupo *et al.* 2020; Table 1; Fig. 6). CDF transporters (including *SlZnT1*, *SlZnT2*, and *SlCDF-D*) have been implicated in the export and sequestration of excess Zn, and their upregulation in high Zn environments has been identified as a potential mechanism of Zn tolerance in *S. luteus* (Ruytinx *et al.* 2017). In addition, chelating agents such as fungal hydrophobins act to bind and neutralize excess metals (Ferrol *et al.* 2016) and upregulation of these genes in high Zn environments is likely another mechanism of Zn tolerance in *S. luteus.* Notably, the Zn-tolerant isolates showed no differences in candidate gene expression when exposed to Zn addition, suggesting the amount of Zn added in our experiment was not enough to induce large responses in the tolerant isolates. Future studies should consider including exposure to multiple levels of Zn to ensure that tolerant isolates are being amply stressed.

Even though our results make significant contributions for understanding the mechanisms of fungal Zn tolerance, this study has some limitations that preclude unveiling additional processes potentially involved in tolerating high Zn. Specifically, ZIP transporters have been shown to have a time sensitive response to excess Zn, with downregulation lasting even only a few hours (Coninx *et al.* 2019). Since we extracted RNA after more than 1 week under Zn stress, the expression profile we captured here reflects an adjusted equilibrium and not immediate responses. Also, fungi most likely deal with excess levels of Zn in both acute and prolonged interactions so future work should examine the differences in the immediate responses of tolerant and sensitive *S. luteus* isolates to excess Zn. Another limitation pertains to the available *S. luteus* reference genome annotation that is far from complete, with well over 50% of genes missing a predicted function. This means that we likely missed important gene categories involved in Zn tolerance. Lastly, epigenetic regulation may contribute to Zn tolerance in *S. luteus* and could provide explanations for transcriptional regulation or lack of thereof. Future studies should rely on an improved reference genome annotation, use higher metal concentrations to assess stress response in tolerant isolates, and include epigenetics, for example, by using whole genome bisulfide sequencing before and after applying Zn treatments.

## Conclusion

In conclusion, our study unveils mechanisms of *S. luteus* Zn tolerance and contributes to the understanding of how fungi can withstand metal toxicity. We found *S. luteus* displays constitutive differences and environmentally driven Zn responses. We also explored expression patterns of genes previously implicated in *S. luteus* metal tolerance and uncovered new signaling genes that potentially contribute to Zn tolerance in the species. Further research can address metal responses of mycorrhizal fungi in conjunction with their obligate plant partners, allowing further understanding of the mechanisms of metal tolerance in a more ecologically relevant context.

## Supplementary Material

jkae156_Supplementary_Data

## Data Availability

The sequence data generated and used in this study are available in the Sequence Read Archive (BioProjects PRJNA972009–PRJNA972026 and PRJNA972046–PRJNA972086). All analytical pipelines are available at https://github.com/ahsmith22/SluteusRNA. Supplemental material available at G3 online.
