# Supplementary material for: Comparative transcriptomics provides insights into molecular mechanisms of zinc tolerance in the ectomycorrhizal fungus Suillus luteus
Source: G3 (Bethesda). 2024 Jul 13;14(9):jkae156. doi: 10.1093/g3journal/jkae156 (PMC11373636; doi:10.1093/g3journal/jkae156)
Supplement: jkae156_Supplementary_Data [file jkae156_supplementary_data.zip › Supplemental_Material_G3-2024-405207.docx]

**Smith et al. “Comparative transcriptomics provides insight into molecular mechanisms of zinc tolerance in the ectomycorrhizal fungus *Suillus luteus*”**

**Supplementary Information**

Comparative transcriptomics provides insight into molecular mechanisms of zinc tolerance in the ectomycorrhizal fungus *Suillus luteus*

Alexander Smith^1^, Jessica Fletcher^1^, Janne Swinnen², Karl Jonckheere², Anna Bazzicalupo^³^, Hui-Ling Liao^4,5^, Greg Ragland^1^, Jan Colpaert^6^, Anna Lipzen^7^, Sravanthi Tejomurthula^7^, Kerrie Barry^7^, Igor Grigoriev^7,8^, Joske Ruytinx², Sara Branco^1^

1. Department of Integrative Biology, University of Colorado Denver, Denver CO, USA
2. Research Groups Microbiology and Plant Genetics, Vrije Universiteit Brussel
3. Comparative Fungal Biology, Royal Botanic Gardens, Kew, Richmond, United Kingdom
4. Soil, Water and Ecosystem Sciences Department, University of Florida, Gainesville, Florida, USA
5. North Florida Research and Education Center, The University of Florida, Quincy, Florida, USA
6. Centre for Environmental Sciences, Hasselt University, Hasselt, Belgium
7. DOE Joint Genome Institute, Lawrence Berkeley National Laboratory, Berkeley, CA, USA
8. Department of Plant and Microbial Biology, University of California Berkeley, Berkeley, California, USA

**Supplementary Information:**

**Supplementary Figures**

**Figure S1 –** WGCNA clustering analyses. Dendrograms of samples (A) before and (B) after exclusion of outliers HZHCN and HZHCP.

**Figure S2 -** WGCNA Soft threshold picking. A range of powers were screened for (A) R^2^ and (B) mean connectivity. Soft power of 16 was selected for an R^2^ > 0.8 and minimal mean connectivity according to best practices.

**Figure S3 -** WGCNA hierarchical clustering dendrogram and heatmap of module eigengene relatedness. In the heat map, columns and row represent module eigengenes by color. Blue represents low adjacency (the modules are negatively correlated), while red represents high adjacency (modules are positively correlation).

**Figure S4** – Volcano plots depicting differentially expressed genes across Zn treatment for each of the studied *S. luteus* isolates. Left – isolates from non-contaminated site; right – isolates from the non-contaminated isolate. Blue – significantly downregulated genes; red – significantly upregulated genes.

**Figure S5** –WGCNA results. (A) Hierarchical clustering gene dendrogram. Color underneath the dendrogram shows the module assignment. Grey module contains all unassigned genes. (B) Bar graph representing the number of genes in each module.

**Figure S6** – WGCNA intermodular gene set enrichment analyses (GSEA). Enrichment plots for gene sets uncovered in analysis of genes ranked by Zn significance (A-C), or EC_50_ significance (D-F). Zn significance: (A) Metal ion binding (B) Metal ion transport and (C) Intracellular signalling cascade. EC_50_ significance (D) Metal ion binding (E) Cation transport (F) Oxidoreductase activity

**Figure S7** – Correlation matrices of the normalized counts for all RNAseq samples in the control (left; n=30) and high Zn conditions (right; n=25).

**Supplementary Tables**

**Table S1** - *S. luteus* isolates included in the experiment, including collection year, Zn tolerance, and location of origin.

**Table S2 –** Short Read Archive Database codes for all samples.

**Table S3 -** Sample raw and filtered read counts.

**Table S4** - Most positively and negatively differentially expressed genes and genes expressed in response to Zn treatment.

**Table S5** – GO terms enriched in the turquoise module (found by WGCNA to be significantly associated with Zn treatment).

**Table S6** – Transcription factors found in WGCNA modules significantly associated with Zn treatment or EC_50._

**Table S7** – Gene set enrichment analyses results for intermodular analysis of genes significantly associated with Zn treatment or EC_50_ in WGCNA analysis.

See GitHub (https://github.com/ahsmith22/SluteusRNA) for commands used in analyses.

**Supplementary Figures**

**
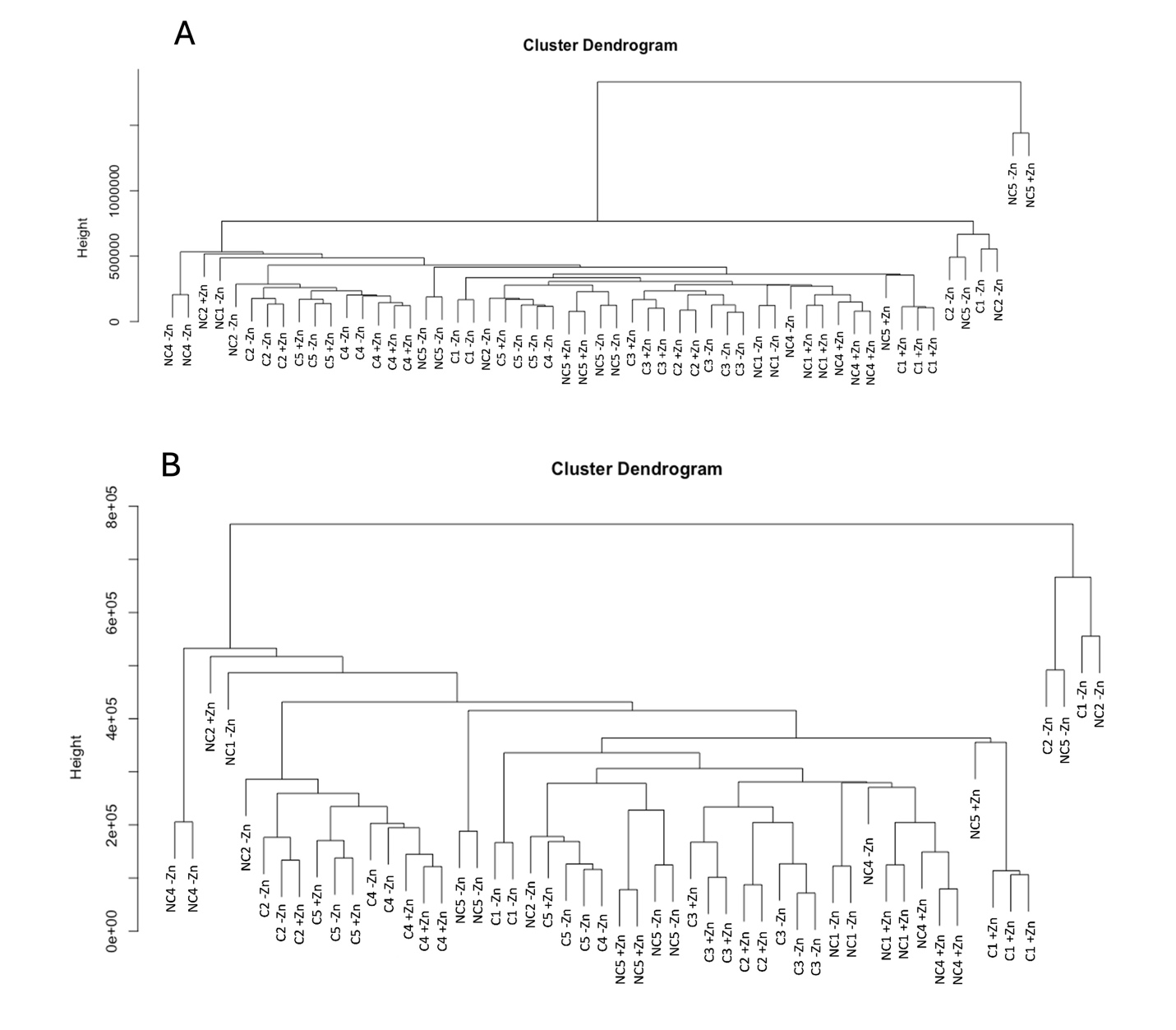
**

**Figure S1 –** WGCNA clustering analyses. Dendrograms of samples (A) before and (B) after exclusion of outliers NC5 -Zn and NC5 +Zn.

**
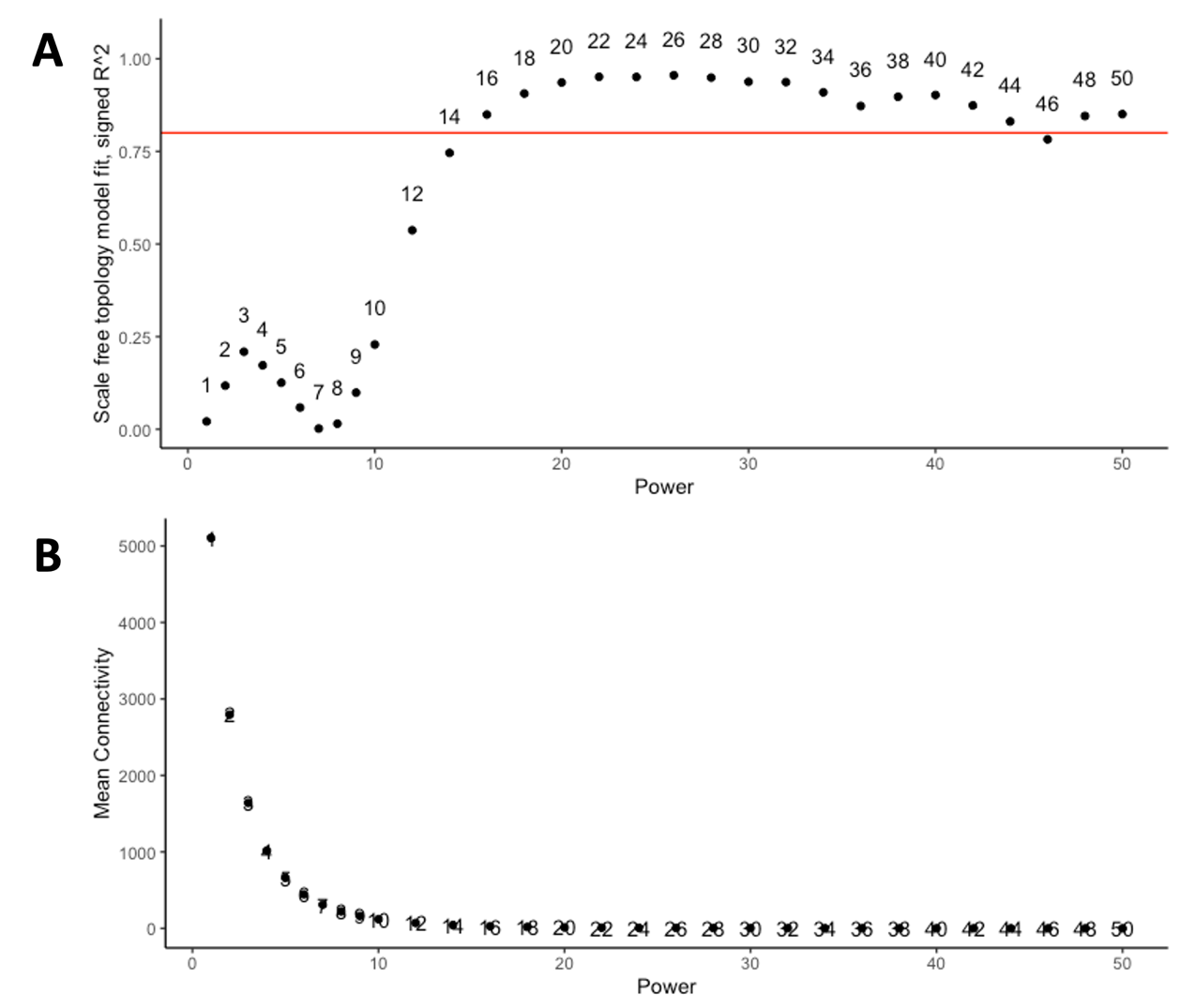
**

**Figure S2 -** WGCNA Soft threshold picking. A range of powers were screened for (A) R^2^ and (B) mean connectivity. Soft power of 16 was selected for an R^2^ > 0.8 and minimal mean connectivity according to best practices.

**
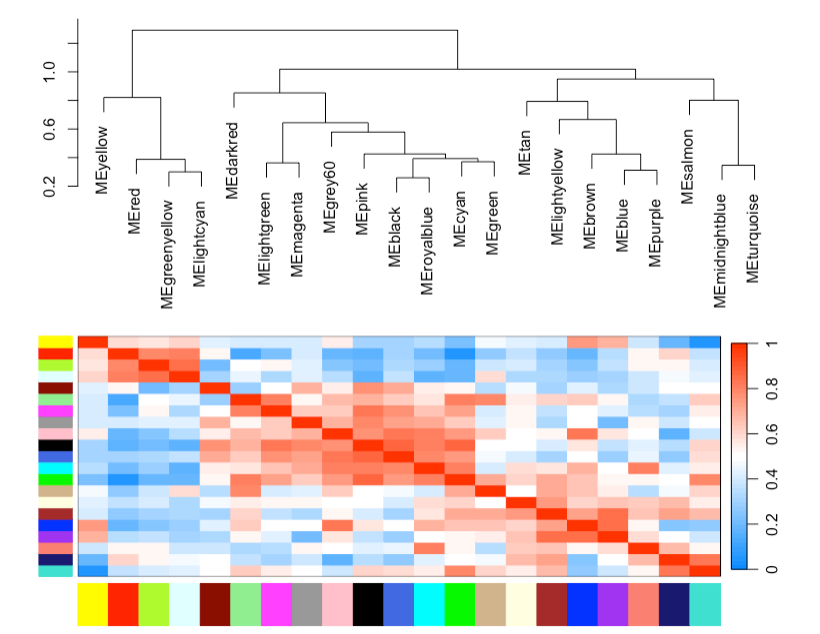
**

**Figure S3 -** WGCNA hierarchical clustering dendrogram and heatmap of module eigengene relatedness. In the heat map, columns and row represent module eigengenes by color. Blue represents low adjacency (the modules are negatively correlated), while red represents high adjacency (modules are positively correlation).


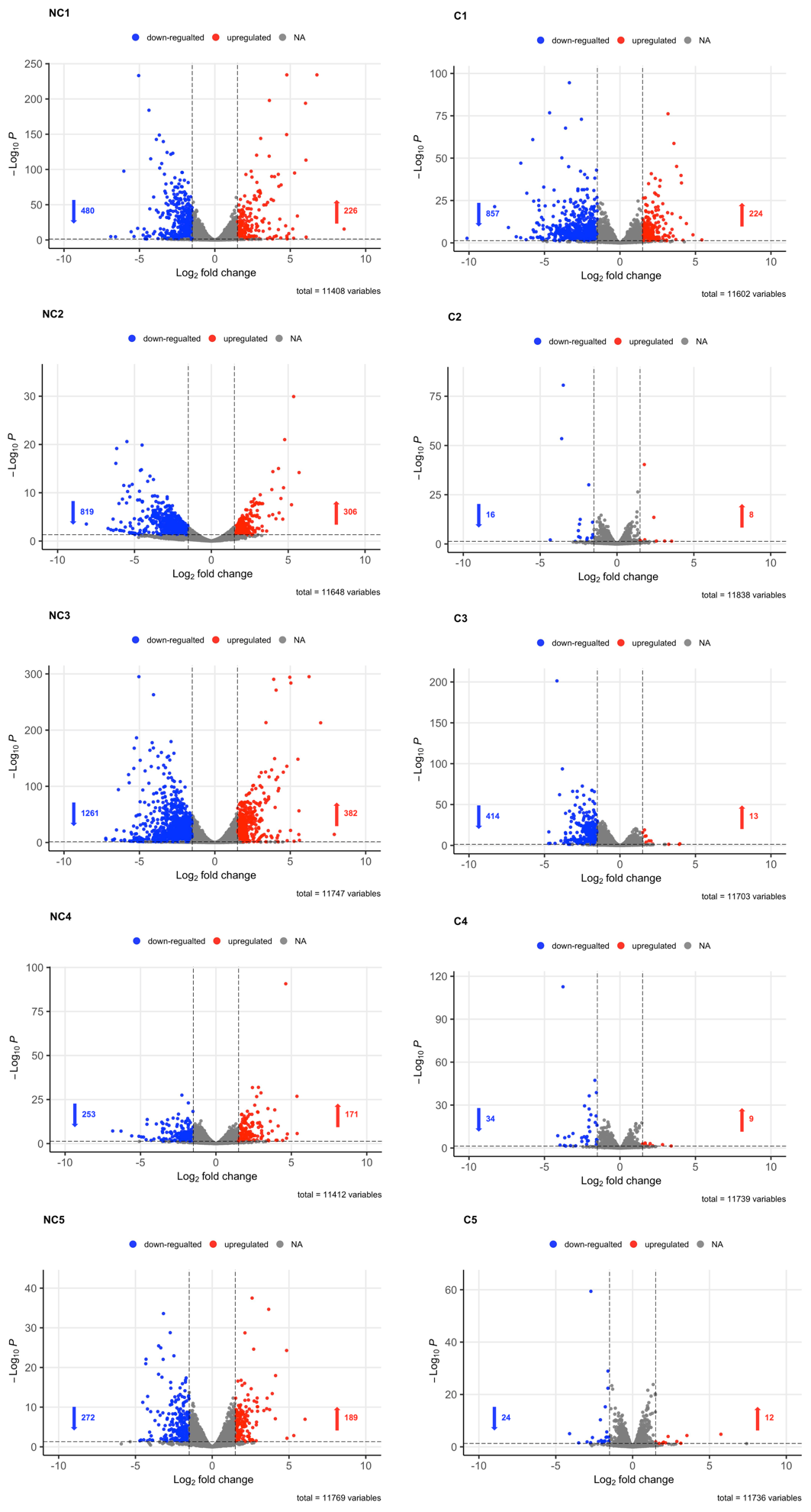


**Figure S4** – Volcano plots depicting differentially expressed genes across Zn treatment for each of the studied *S. luteus* isolates. Left – isolates from non-contaminated site; right – isolates from the contaminated isolate. Blue – significantly downregulated genes [p<0.05, L_2_FC <= -1.5]; red – significantly upregulated genes[p<0.05, L_2_FC >= 1.5]


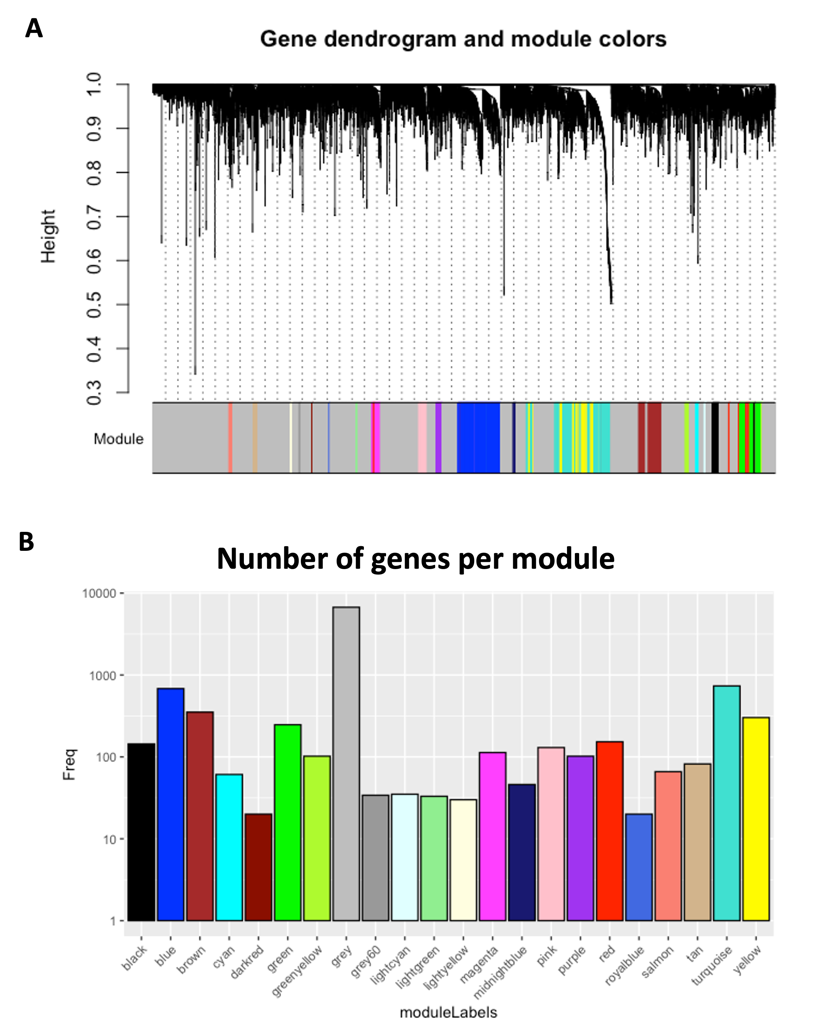


**Figure S5** –WGCNA results. (A) Hierarchical clustering gene dendrogram. Color underneath the dendrogram shows the module assignment. Grey module contains all unassigned genes. (B) Bar graph representing the number of genes in each module.


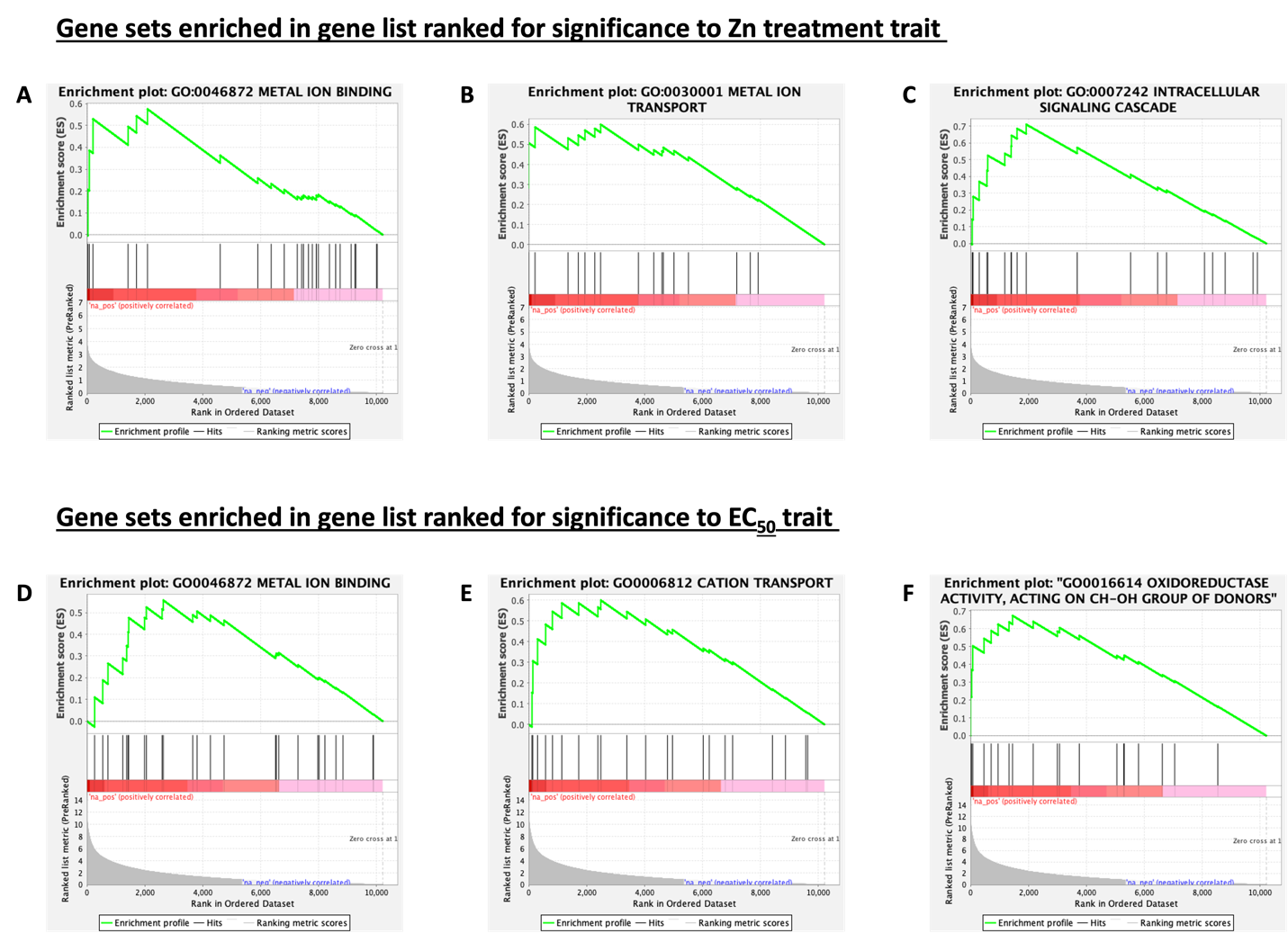


**Figure S6** – WGCNA intermodular gene set enrichment analyses (GSEA). Enrichment plots for gene sets uncovered in analysis of genes ranked by Zn significance (A-C), or EC_50_ significance (D-F). Zn significance: (A) Metal ion binding (B) Metal ion transport and (C) Intracellular signalling cascade. EC_50_ significance (D) Metal ion binding (E) Cation transport (F) Oxidoreductase activity

**
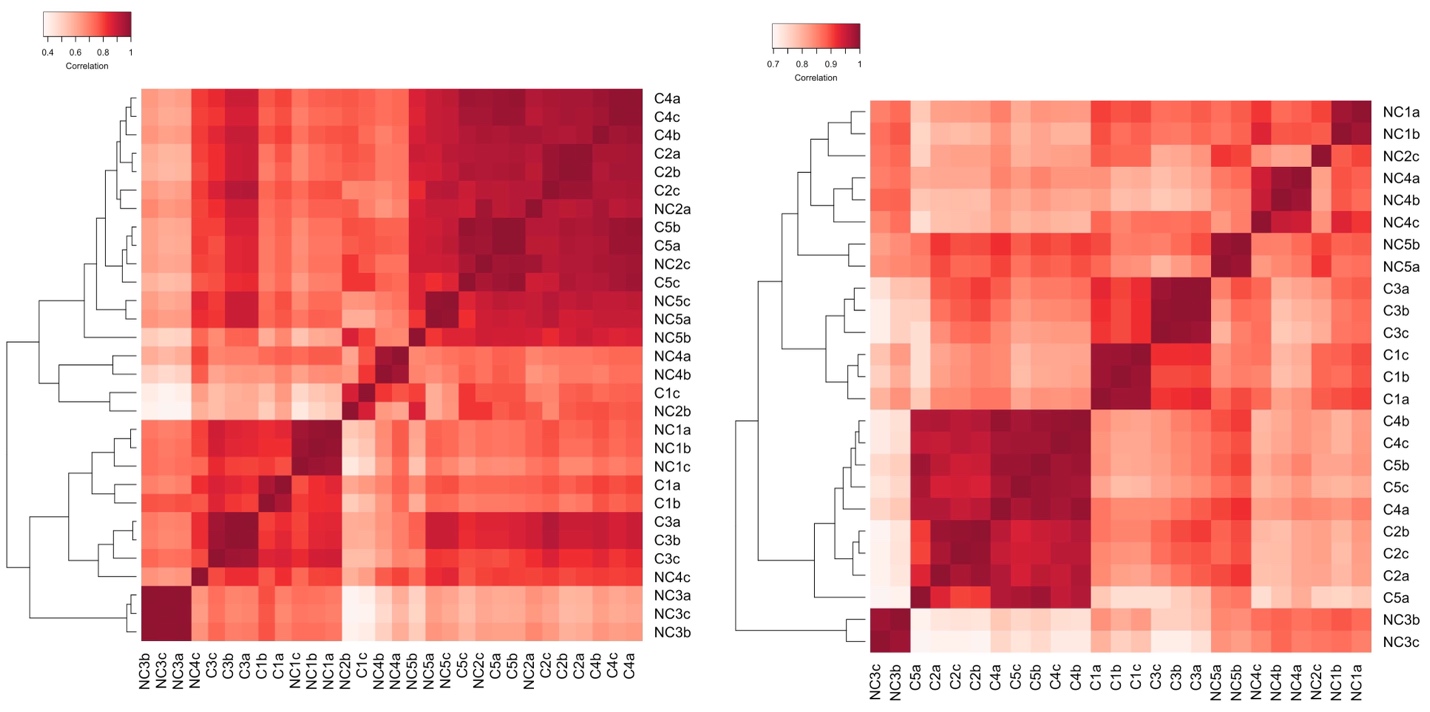
**

**Figure S7** – Correlation matrices of the normalized counts for all RNAseq samples in the control (left; n=30) and high Zn conditions (right; n=25).
